# Willingness to pay for Social Health Insurance and associated factors among Public Civil Servants in Ethiopia: A systematic review and meta-analysis

**DOI:** 10.1371/journal.pone.0293513

**Published:** 2024-02-09

**Authors:** Abdene Weya Kaso, Girma Worku Obsie, Berhanu Gidisa Debela, Abdurehman Kalu Tololu, Esmael Mohammed, Habtamu Endashaw Hareru, Daniel Sisay, Gebi Agero, Alemayehu Hailu

**Affiliations:** 1 Department of Public Health, College of Health Science, Arsi University, Asella, Ethiopia; 2 School of Public Health, College of Health Science and Medicine, Dilla University, Dilla, Ethiopia; 3 Bokoji Primary Hospital, Oromia Health Bureau, Bokoji, Ethiopia; 4 Bergen Centre for Ethics and Priority Setting, Department of Global Public Health and Primary Care, University of Bergen, Bergen, Norway; 5 Faculty of Health and Social Science, Section for Global Health and Rehabilitation, Western Norway University of Applied Sciences, Bergen, Norway; University for Development Studies, GHANA

## Abstract

**Background:**

The provision of equitable and accessible healthcare is one of the goals of universal health coverage. However, due to high out-of-pocket payments, people in the world lack sufficient health services, especially in developing countries. Thus, many low and middle-income countries introduced different prepayment mechanisms to reduce large out-of-pocket payments and overcome financial barriers to accessing health care. Though many studies were conducted on willingness to pay for social health insurance in Ethiopia, there is no aggregated data at the national level. Therefore, this systematic review and meta-analysis aimed to estimate the pooled magnitude of willingness to pay for social health insurance and its associated factors among public servants in Ethiopia.

**Method:**

Studies conducted before June 1, 2022, were retrieved from electronic databases (PubMed/Medline, Science Direct, African Journals Online, Google Scholar, and Web of Science) as well as from Universities’ digital repositories. Data were extracted using a data extraction format prepared in Microsoft Excel and the analysis was performed using STATA 16 statistical software. The quality of the included studies was assessed using the Newcastle-Ottawa Scale for cross-sectional studies. To evaluate publication bias, a funnel plot, and Egger’s regression test were utilized. The study’s heterogeneity was determined using Cochrane Q test statistics and the I^2^ test. To determine the pooled effect size, odds ratio, and 95% confidence intervals across studies, the DerSimonian and Laird random-effects model was used. Subgroup analysis was conducted by region, sample size, and publication year. The influence of a single study on the whole estimate was determined via sensitivity analysis.

**Result:**

To estimate the pooled magnitude of willingness to pay for the Social Health insurance scheme in Ethiopia, twenty articles with a total of 8744 participants were included in the review. The pooled magnitude of willingness to pay for Social Health Insurance in Ethiopia was 49.62% (95% CI: 36.41–62.82). Monthly salary (OR = 6.52; 95% CI:3.67,11.58), having the degree and above educational status (OR = 5.52; 95%CI:4.42,7.17), large family size(OR = 3.69; 95% CI:1.10,12.36), having the difficulty of paying the bill(OR = 3.24; 95%CI: 1.51, 6.96), good quality of services(OR = 4.20; 95%CI:1.97, 8.95), having favourable attitude (OR = 5.28; 95%CI:1.45, 19.18) and awareness of social health insurance scheme (OR = 3.09;95% CI:2.12,4.48) were statistically associated with willingness to pay for Social health insurance scheme.

**Conclusions:**

In this review, the magnitude of willingness to pay for Social Health insurance was low among public Civil servants in Ethiopia. Willingness to pay for Social Health Insurance was significantly associated with monthly salary, educational status, family size, the difficulty of paying medical bills, quality of healthcare services, awareness, and attitude towards the Social Health Insurance program. Hence, it’s recommended to conduct awareness creation through on-the-job training about Social Health Insurance benefit packages and principles to improve the willingness to pay among public servants.

## Introduction

World Health Organization (WHO) introduced universal health coverage to ensure equitable and accessible healthcare without financial barriers at the point of service delivery [1, 2]. However, significant proportions of people all over the world suffer due to a lack of access to basic healthcare services. Globally, around 150 million people in developing countries face catastrophic financial expenditure each year. In addition, around 100 million people were pushed into poverty as a result of out-of-pocket (OOP) expenditure at service delivery points [3, 4]. In many low and middle-income countries(LMIC), healthcare services were underfinanced and relied on OOP health expenditure as the major source of healthcare financing [5]. According to the WHO 2012 report, the share of OOP measured was 48% of total expenditure in Sub-Saharan African countries whereas it is around 14% in countries with higher incomes [2]. Moreover, high OOP, inadequate healthcare financing, and the absence of prepayment methods for formal sectors were the major challenges to the health delivery system in Ethiopia for a decade [6, 7]. In Ethiopia, OOP accounted for 34% of the total healthcare expenditures and exposed a significant proportion of households and formal sector workers to financial hardship for many years [8–10]. Thus, the Ethiopian government considered the Social Health Insurance (SHI) as a method to mobilize and pool additional resources for health from formal sector workers through a payroll-based system in 2011. According to the plan, pensioners will contribute 1% of their monthly salary while active employees pay a monthly premium of 3%. This facilitates the provision of affordable healthcare services and aids formal workers in managing healthcare costs. Despite the government’s plan to fully implement SHI by 2014, it is not well-practiced and implemented due to public workers’ opposition to the scheme [11, 12]. Previous studies conducted in different parts of the country indicate different levels of willingness to pay (WTP) for the SHI scheme. For instance, the civil servants’ willingness to pay was 69.8% in the Derba Berhan [13], 62% in the Gondar town [14], and 66.6% in Northwest Ethiopia [15]. Moreover, previous studies revealed that 74.9% of the workers in Mekelle [16], 28.7% in Addis Ababa [17], and 74.4% in the SNNPR [18] were willing to pay for the SHI scheme. Studies have shown that factors that affect WTP for the SHI include socioeconomic factors, healthcare relatedness, attitude, and knowledge of the SHI scheme [13, 14, 17, 19–21]. Despite different studies conducted in Ethiopia; there has been a wide discrepancy and lack of nationally representative data on WTP for the SHI scheme. Therefore, this systematic review and meta-analysis aimed to estimate the pooled magnitude of WTP for the SHI scheme and associated factors among public servants in Ethiopia. The findings of this systematic review and meta-analysis will help to combine previous findings and demonstrate the effects of relevant variables on WTP for the SHI scheme. Furthermore, the presence of aggregated information is essential to assist policymakers, health providers, and the government in planning and making evidence-based decisions.

## Method and material

### Study protocol and registration

To determine Ethiopian public employees’ pooled WTP for the SHI, a systematic review and meta-analysis were conducted in accordance with PRISMA (Preferred Reporting Items for Systematic Reviews and Meta-Analysis Statement) criteria. A revised PRISMA 2020 guideline was used to create the report of this systematic review and meta-analysis [22]. The protocol for this systematic review and meta-analysis was registered at PROSPERO (registration ID = CRD42023457003).

### Search strategy

Two reviewers (BGD and HEH) conducted exhaustive and comprehensive searches independently on electronic databases such as PubMed/Medline, Scopus, African Journals Online, Google Scholar, and Web of Science for articles until June 1, 2022. To obtain the articles, we used keywords such as “Willingness to pay”, “Demand”, “Acceptance”, “Social Health Insurance”, “Associated factors”, “Determinants”, “Predictors”, “Public Civil Servants” and “Ethiopia”. We also used Boolean operators "AND”/"OR" to combine terms for the search of articles. The full electronic search strategy for PubMed is shown (S1 Table). In addition, we searched the Ethiopian University electronic library for unpublished studies. Furthermore, when an article lacked sufficient data, we contacted the corresponding authors via email.

### Inclusion and exclusion criteria

All English-language observational studies (cross-sectional, case-control, and cohort study designs) conducted since 2010 (i.e. the period the Ethiopian government launched the insurance scheme in the healthcare financing reform) and reported the level of WTP for the SHI scheme and its associated factors among public workers or Civil servants in Ethiopia were taken into consideration. However, studies that did not report the level of WTP for the SHI program, publications without full texts, qualitative studies, systematic reviews, editorials, conference reports, commentaries, and letters were eliminated from the study.

### Outcome of interest

The primary outcome of the study was the pooled magnitude of willingness to pay for the SHI scheme in Ethiopia. The WTP for the SHI scheme was defined as the motive of public workers to pay 3% or 1% of their salary to enroll in the Social health insurance scheme [23]. The secondary outcome of the study includes socioeconomic and demographic factors (educational status, family size, and monthly salary); perceived quality of healthcare services, the ability to pay the bill, and awareness of the SHI scheme that determine the willingness to pay for the SHI scheme.

### Study selection

Two reviewers reviewed the studies that met the inclusion and exclusion criteria’s. We conducted a two-level screening approach to evaluate the identified studies from different sources. First, the titles and abstracts were reviewed for the chosen studies and irrelevant titles and abstracts were removed. Second, the full-text articles were collected and reviewed to ensure that they were eligible. Any inconsistencies between the two reviewers were resolved by discussions with the primary investigator (AWK) to reach an agreement. The studies were extracted from various databases and imported into Endnote reference management software version x7.1, where duplicates were removed. The PRISMA flow diagram was used to summarize the study selection method.

### Data extraction

Two authors (BGD and HEH) independently extracted the general characteristics of the studies using a data extraction format designed in Microsoft Excel. The data abstraction format contains the general information of the studies such as the author’s name, year of publication, study design, response rate, region, sample size, and magnitude of WTP for the SHI program. In addition, a two-by-two table was prepared in the data extraction format for extracting the frequency of factors associated with workers’ WTP for the SHI scheme. Factors associated with WTP for the SHI scheme were considered for the systematic review and meta-analysis if it was reported as a factor associated with at least three studies.

### Quality assessment

The qualities of primary studies were assessed using the Newcastle-Ottawa Scale. The Newcastle-Ottawa Scale (NOS) has three main domains and is graded out of ten points (stars): the first section evaluates the methodological quality (i.e. representativeness of the sample, sampling technique, response rate, and ascertainment of exposure) of each study and weighs five stars. Besides, the second section considers the comparability (i.e. adjustments for relevant predictors/risk factors/confounders) of the study and takes two stars while the third section evaluates the study’s outcome with regard to statistical analysis (3 stars) [24]. The quality of the included articles was independently appraised by two reviewers (BGW and HEH). The revised Newcastle–Ottawa quality rating scale for cross-sectional research awarded a maximum score of 10 to cross-sectional studies. Even though there is no clear cut-off point using the Newcastle–Ottawa Scale, the Quality of studies was classified as very good (9–10 points), good(7–8 points), satisfactory(5–6 points), and unsatisfactory(0–4 points) based on previous study [25]. Finally, we include a study that scored satisfactory and above in the meta-analysis. The discrepancy among reviewers during the quality assessment was solved by taking the mean score of their assessment results.

### Data analysis

We used Microsoft Excel spread sheets for data collection and imported them into STATA version 16 for analysis. We utilized Cochran’s Q and I^2^ statistics to assess the heterogeneity among the studies analyzed. The I^2^ statistical test calculates the proportion of variance in effect estimates that can be attributed to heterogeneity rather than sampling error. We determined the presence of low, medium, and high heterogeneity using the I^2^ test values of 25%, 50%, and 75%, respectively [26, 27]. An I^2^>50% or p<0.1 indicated significant heterogeneity, for which the DerSimonian and Laird random-effects model was utilized [28]. In addition, we evaluated the publication bias through visual inspections of the asymmetry of the funnel plots and using Begg and Egger’s test, with a value of less than 5% as a cut-off point to declare the presence of publication bias. We performed subgroup analysis by study region, sample size, and publication year to reduce the random variations between the primary study’s point estimates. The pooled odds ratio with their 95% confidence intervals was estimated to determine the association between predictors’ variables and willingness to pay for SHI. We also conducted a leave-one-out sensitivity analysis to assess the influence of a single study on the proportion of public employees’ willingness to pay for the SHI scheme.

## Result

### Study selection

**Fig 1:**
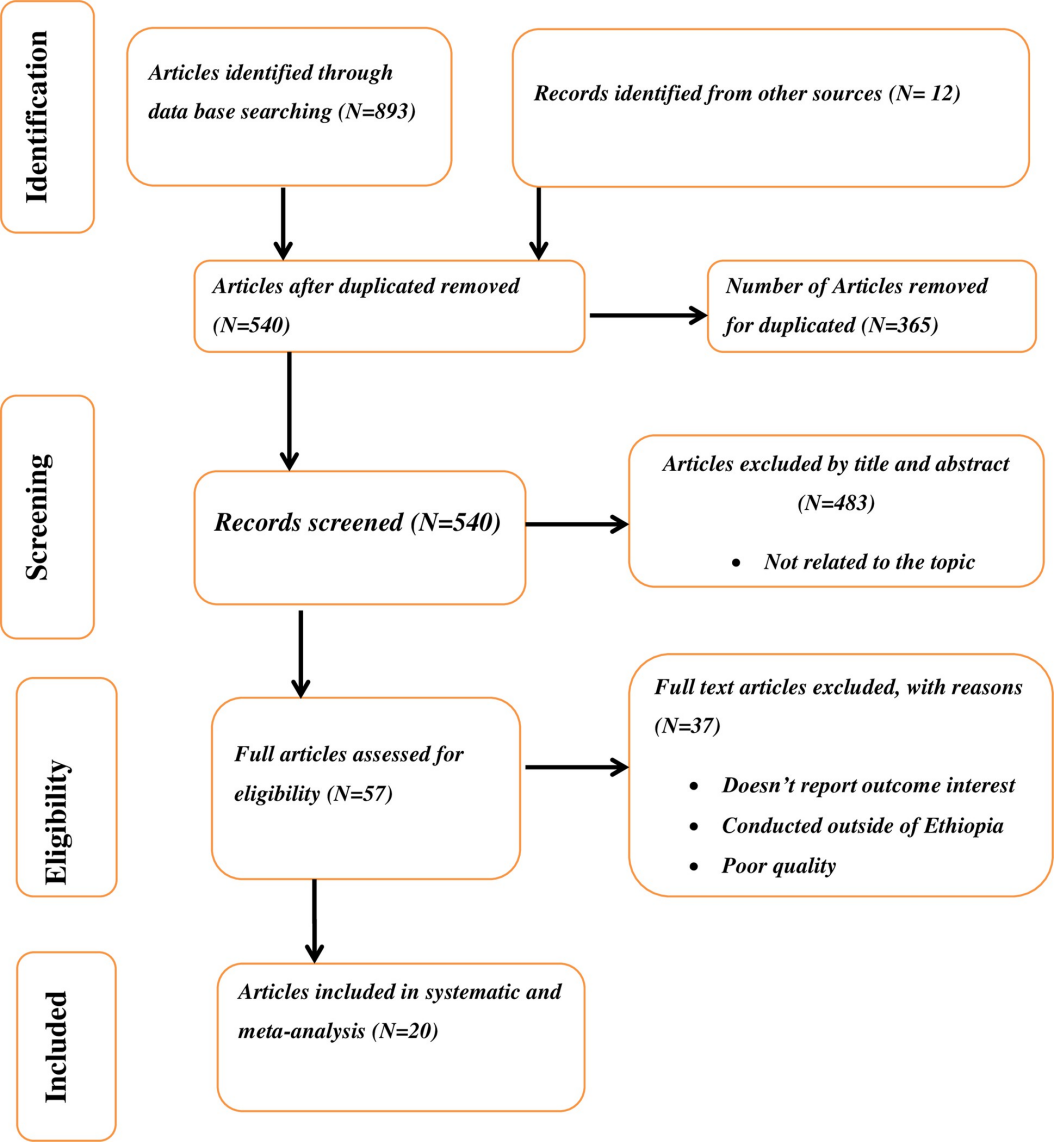
Study flow diagram for a systematic review and meta-analysis of willingness to pay for the SHI scheme and its associated factors in Ethiopia, 2022.

The process and the result of record identifications were indicated in the PRISMA flow diagram (Fig 1). We identified a total of 893 records through a comprehensive search of electronic databases and 12 records from other sources. Of these articles identified, we excluded 365 studies due to duplication. The remaining 540 studies titles and abstract were screened and 483 irrelevant records were excluded. After that, the remaining 57 articles were reviewed for eligibility. Among the potentially eligible studies, 37 full-text articles were removed for not being conducted in Ethiopia and reported outcomes of the interest. Then, 20 articles were included to assess the pooled magnitude of WTP pay for the SHI scheme and its associated factors among public servants in Ethiopia.

### Characteristics of included study

As indicated in Table 1, 20 cross-sectional studies with a total sample size of 8744 workers were included in this systematic review and meta-analysis. The studies were conducted from 2014 to 2022 and the retrieved studies were from the Southern Nations, Nationalities, and People Region (SNNPR) [18, 29], Amhara [13–15, 21, 30–32], Addis Ababa [17, 19, 20, 33–35], Harar [36], Oromia [37] and from Tigray [16, 38, 39]. The sample size of the study ranged from 218 to 843 participants. The highest magnitude of willingness to pay for SHI was reported from the Harar region, 89.5% [36], and the lowest was from the Addis Ababa city administration, 17% [19] (Table 1).

**Table 1:** Descriptive summary of studies reporting the willingness to pay for social health insurance in Ethiopia, 2022.

Authors	Region	year	sample size	Type of outcome	Prevalence	Study design	NOQS
Abebaw B. et al	Amhara	2020	421	WTP	69.8(65.42,74.19)	Cross-sectional	8
Zemene. et al	Amhara	2020	541	WTP	32(28.07,35.93)	Cross-sectional	7
Tewele A. et al.	Tigray	2020	408	WTP	74.9(70.69,79.11)	Cross-sectional	7
Lasebew Y. et al	AA	2017	409	WTP	17(13.36,20.64)	Cross-sectional	7
Degie FM. et al	Amhara	2021	375	WTP	37.6(32.7,42.5)	Cross-sectional	7
Tesfamichael A. et al	SNNPR	2014	328	WTP	74.4(69.68,79.12)	Cross-sectional	7
Mekonne A. et al	AA	2019	460	WTP	28.7(24.57,32.83)	Cross-sectional	7
Getahun T. et al	Amhara	2021	324	WTP	61.0(55.69,66.31)	Cross-sectional	7
Gessese TA. et al	Tigray	2016	843	WTP	36.0(32.76,39.24)	Cross-sectional	8
Setegn A. et al	Amhara	2021	574	WTP	62.0(58.03,65.97)	Cross-sectional	7
Gidey. et al	Tigray	2019	381	WTP	85.3(81.74,88.86)	Cross-sectional	7
Yeshiwas S. et al	Amhara	2018	488	WTP	66.6(62.42,70.78)	Cross-sectional	7
Mekonnen WN. Et al	Amhara	2019	619	WTP	27.8(24.27,31.33)	Cross-sectional	7
Yonas Hizkiyas	AA	2020	398	WTP	77.1(72.97,81.23)	Cross-sectional	7
Mulatu B. et al	SNNPR	2020	692	WTJ &WTP	6(4.23,7.77)	Cross-sectional	5
Regassa Z. et al	Oromia	2018	280	WTP	58.6(52.83,64.37)	Cross-sectional	7
Hailu et al	Harar	2022	323	WTP	89.5(86.16,92.84)	Cross-sectional	8
Tadele W. et al	AA	2020	368	WTP	24.1(19.73,28.47)	Cross-sectional	7
Obse A. et al	AA	2016	218	WTP	29.0(22.98,35.02)	Cross-sectional	7
Tenaw Y	AA	2017	294	WTP	35.0(29.55,40.45)	Cross-sectional	8

**Note**: AA: Addis Ababa, NOQS, New Ottawa Quality score, SNNPR: Southern Nations, Nationalities, and Peoples, WTJ: Willingness to Join

### Magnitude of willingness to pay for the SHI scheme in Ethiopia

**Fig 2:**
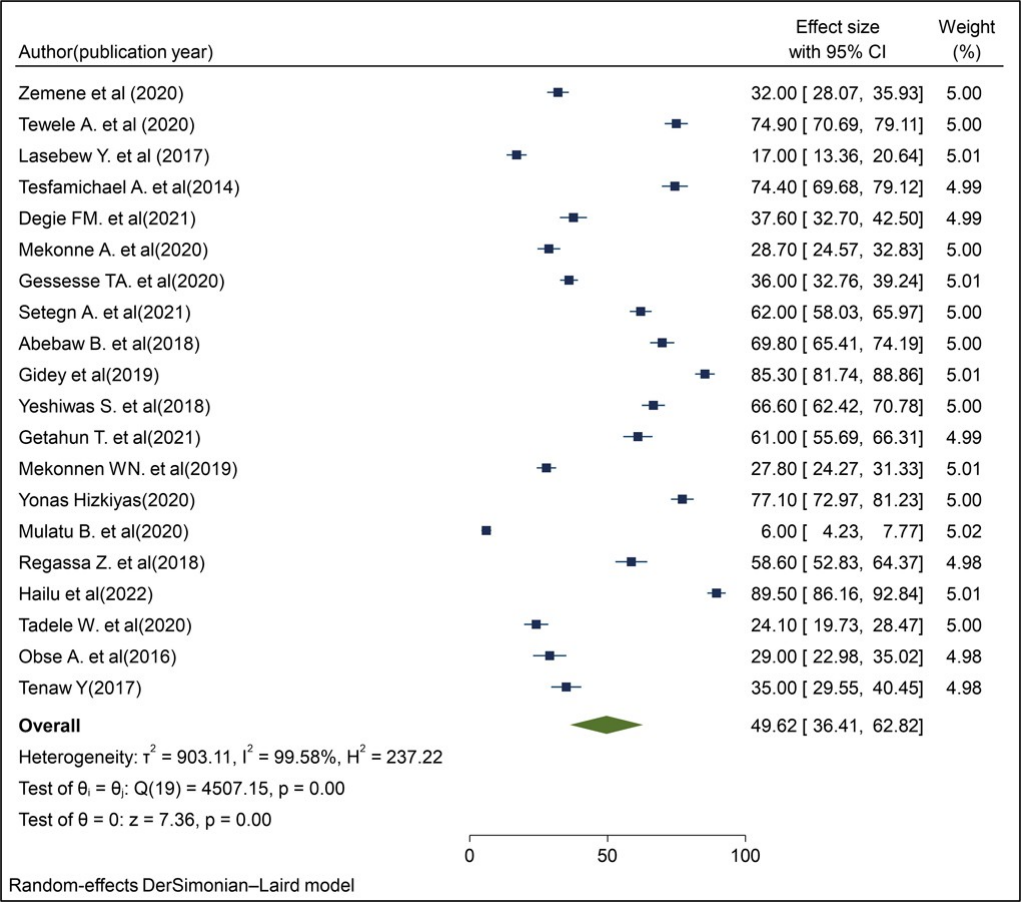
Forest plot for the pooled magnitude of willingness to pay for the SHI scheme among public servants in Ethiopia, 2022.

The magnitude of WTP for the SHI scheme estimate varied among studies with significant heterogeneity (P < 0.001; I^2^ = 99.6%). Thus, we employed a random effect model to estimate the pooled prevalence of WTP for the SHI scheme among workers in Ethiopia. The pooled magnitude of WTP for the SHI scheme in Ethiopia among workers was 49.62% (95% CI: 36.41–62.82) based on the DerSimonian–Laird random-effect model [13–21, 29–39] (Fig 2).

### Publication bias

**Fig 3:**
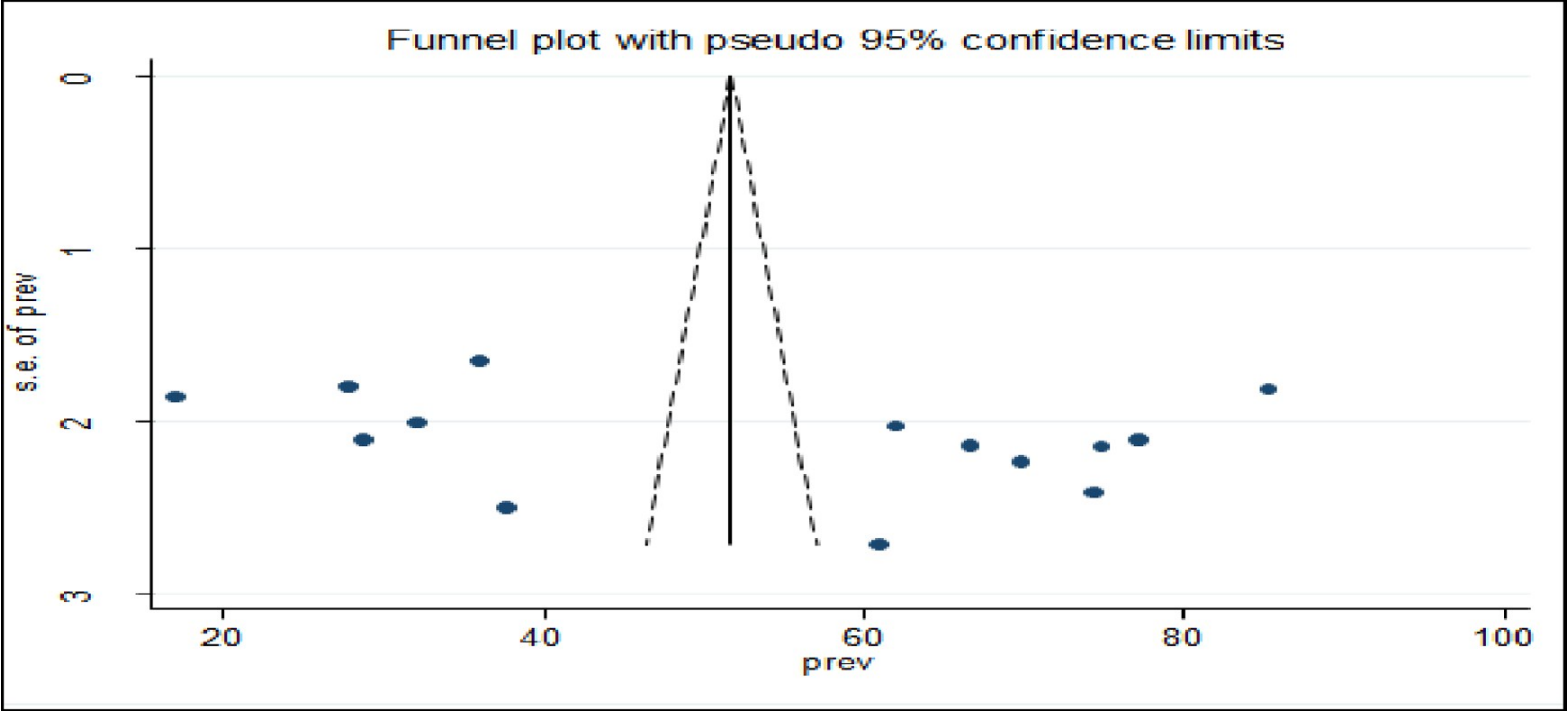
Funnel plot with 95% confidence limits of the pooled magnitude of willingness to pay for SHI among public servants in Ethiopia, 2022.

We used Begg’s and Egger’s test and funnel plot to assess the presence of potential publication bias in the included studies. Visual inspection of the asymmetric funnel plot showed the absence of publication bias, which was statistically supported by Egger’s test (bias coefficient (B) = -6.43(95%CI = − 45.34–32.47; P = 0.732) and Begg’s test (P = 0.846) (Fig 3).

### Subgroup analysis

We performed subgroup analysis in this study by region, sample size, and year of publication. The sub-group analysis revealed that the Harar region had the highest magnitude of WTP for the SHI scheme, with a pooled magnitude of 89.5% (95% CI: 86.16, 92.84%) [36], and the Addis Ababa city administration had the lowest WTP for the SHI scheme, with a magnitude of 35.16% (95% CI: 13.56, 53.96%) [17, 19, 20, 33–35]. The other subgroup analysis was done for the sample size and year of publication of the study. The lowest WTP for the SHI scheme was reported for studies having sample size >400(42.05% (95% CI: 25.62, 58.48%) [13–17, 19, 29, 31, 32, 39], and published after 2018 (49.38% (95% CI: 31.89, 66.87) [14, 16, 17, 20, 21, 29–33, 36, 38, 39] (Table 2).

**Table 2:** Sub-group analysis of the magnitude of willingness to pay for social health insurance in Ethiopia, 2022 (n = 20).

Variables	Subgroup	Number of studies included	Level of WTP(95%CI)	Heterogeneity across studies	
				I^2^ (%)	P-Value
Region	Oromia	1	58.6(52.83,64.37)	0.0	-
	Amhara	7	50.95(37.14,64.76)	98.7	<0.001
	Tigray	3	65.39(33.89,96.89)	99.6	<0.001
	SNNPR	2	40.16(-26.87,107.19)	99.9	<0.001
	Harar	1	89.5(86.16,92.84)	0.0	
	Addis Ababa	6	35.16(16.35,53.96)	99.1	<0.001
Sample size	>400	10	42.05(25.62,58.48)	99.6	<0.001
	<400	10	57.21(41.36,73.06)	99.2	<0.001
Year	>2018	13	49.38(31.89,66.87)	99.7	<0.001
	<2018	7	50.07(31.62, 68.51)	99.1	<0.001

### Sensitivity analysis

Table 3 indicates the sensitivity analysis of WTP for the SHI scheme for each study that omitted one at a time. To identify the potential source of heterogeneity in the analysis, a leave-one-out sensitivity analysis in WTP for SHI scheme civil servants in Ethiopia was conducted. We found that no single study had an effect on the overall WTP for the SHI scheme of workers.

**Table 3:** Sensitivity analysis of included studies for the influence of one study on the overall estimate.

Study excluded	Level of WTP for SHI	95%CI	I^2^ (%)	Q-Value	P-Value
Zemene. et al	50.54	39.20,61.89	99.4	345.27	<0.001
Tewele A. et al.	48.28	37.11,59.45	99.38	354.0	<0.001
Lasebew Y. et al	51.33	40.39, 62.28	99.35	479.27	<0.001
Tesfamichael A. et al	48.31	37.13,59.50	99.39	347.45	<0.001
Degie FM. et al	50.25	38.28,61.67	99.42	372.49	<0.001
Mekonne A. et al	50.72	39.44,61.99	99.39	345.15	<0.001
Gessese TA. et al	50.35	38.92,61.74	99.39	345.48	<0.001
Setegn A. et al	48.96	37.54,60.39	99.41	347.09	<0.001
Abebaw B. et al	48.56	37.26,59.84	99.40	349.50	<0.001
Gidey. et al	47.73	36.90,58.56	99.33	777.50	<0.001
Yeshiwas S. et al	48.72	37.36,60.07	99.40	345.86	<0.001
Getahun T. et al	49.02	37.58,60.45	99.42	346.41	<0.001
Mekonnen WN. Et al	50.77	39.51,62.02	99.38	386.50	<0.001
Yonas Hizkiyas	48.17	37.06,59.28	99.38	355.45	<0.001
Mulatu B. et al	51.93	41.45,42.40	99.16	345.72	<0.001
Regassa Z. et al	49.15	37.68,60.60	99.43	349.46	<0.001
Hailu et al	47.51	36.85,58.16	99.30	156.75	<0.001
Tadele W. et al	50.96	39.79,62.12	99.39	345.05	<0.001
Obse A. et al	50.69	39.41,61.97	99.41	120.94	<0.001
Tenaw Y	50.38	38.99,61.77	99.42	971.71	<0.001

### Factors associated with public workers’ willingness to pay for the SHI scheme in Ethiopia

**Fig 4:**
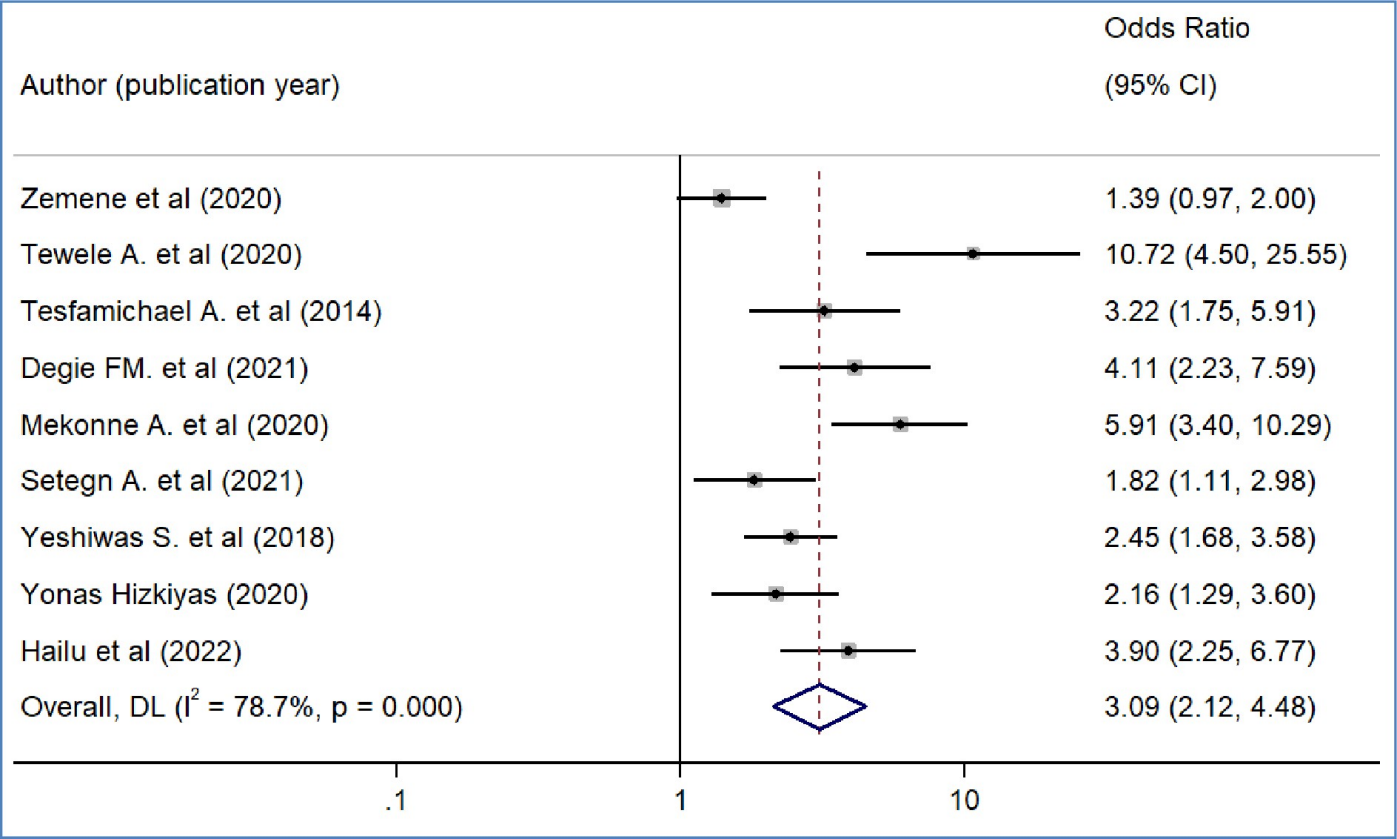
The pooled odds ratio of the association between willingness to pay for SHI and awareness about the SHI program in Ethiopia, 2022.

**Fig 5:**
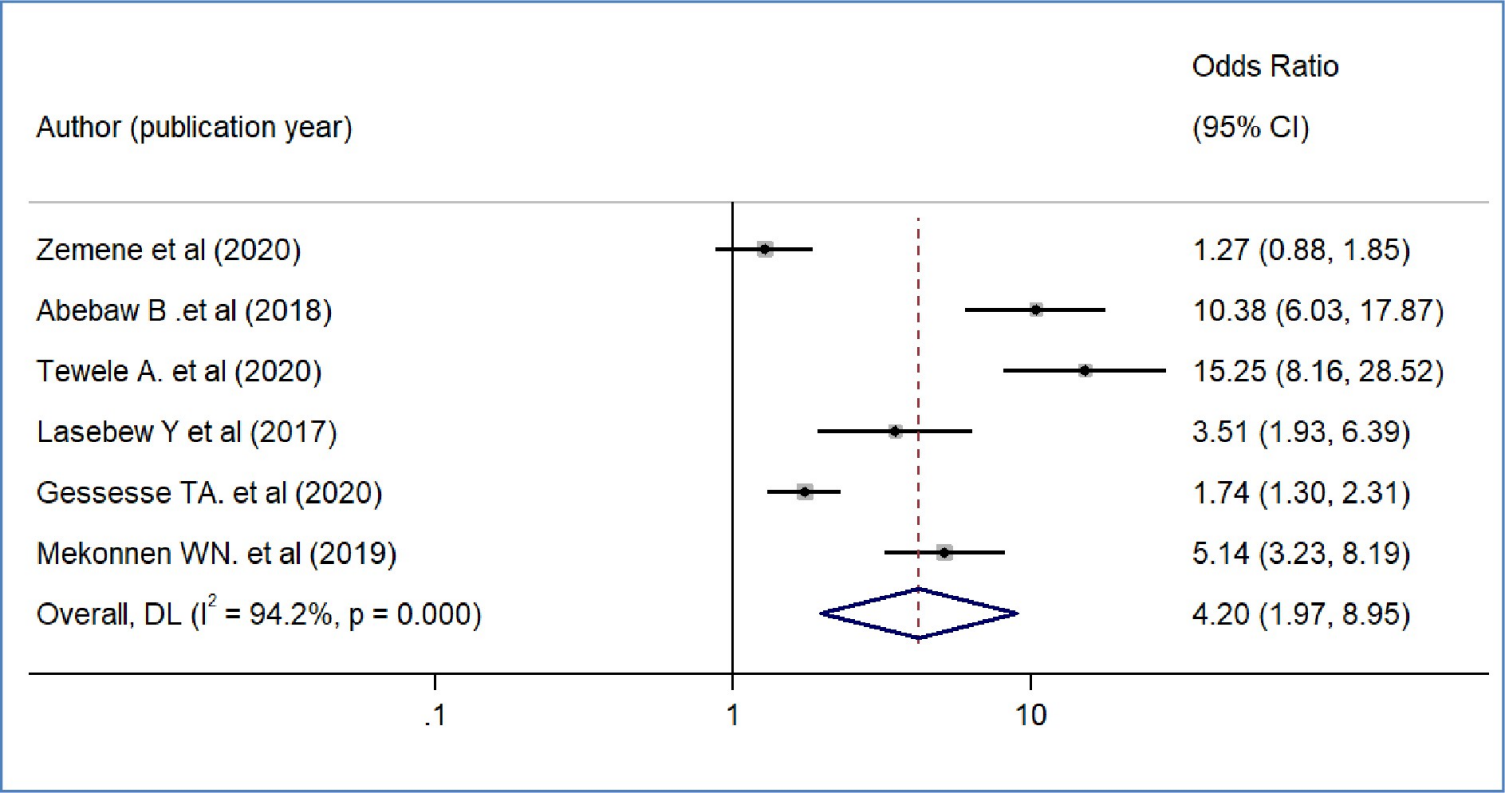
The pooled odds ratio of the association between willingness to pay for SHI and family size in Ethiopia, 2022.

**Fig 6:**
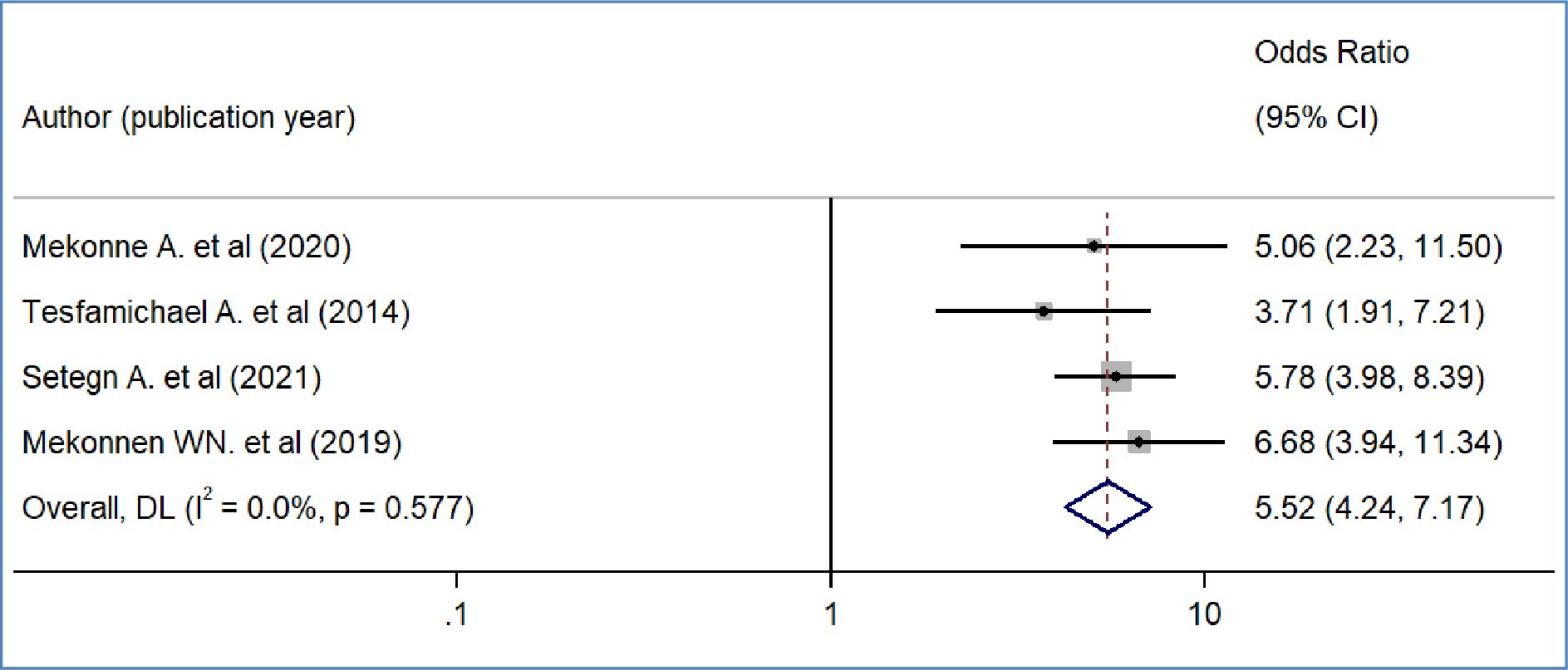
The pooled odds ratio of the association between willingness to pay for SHI and educational status in Ethiopia, 2022.

**Fig 7:**
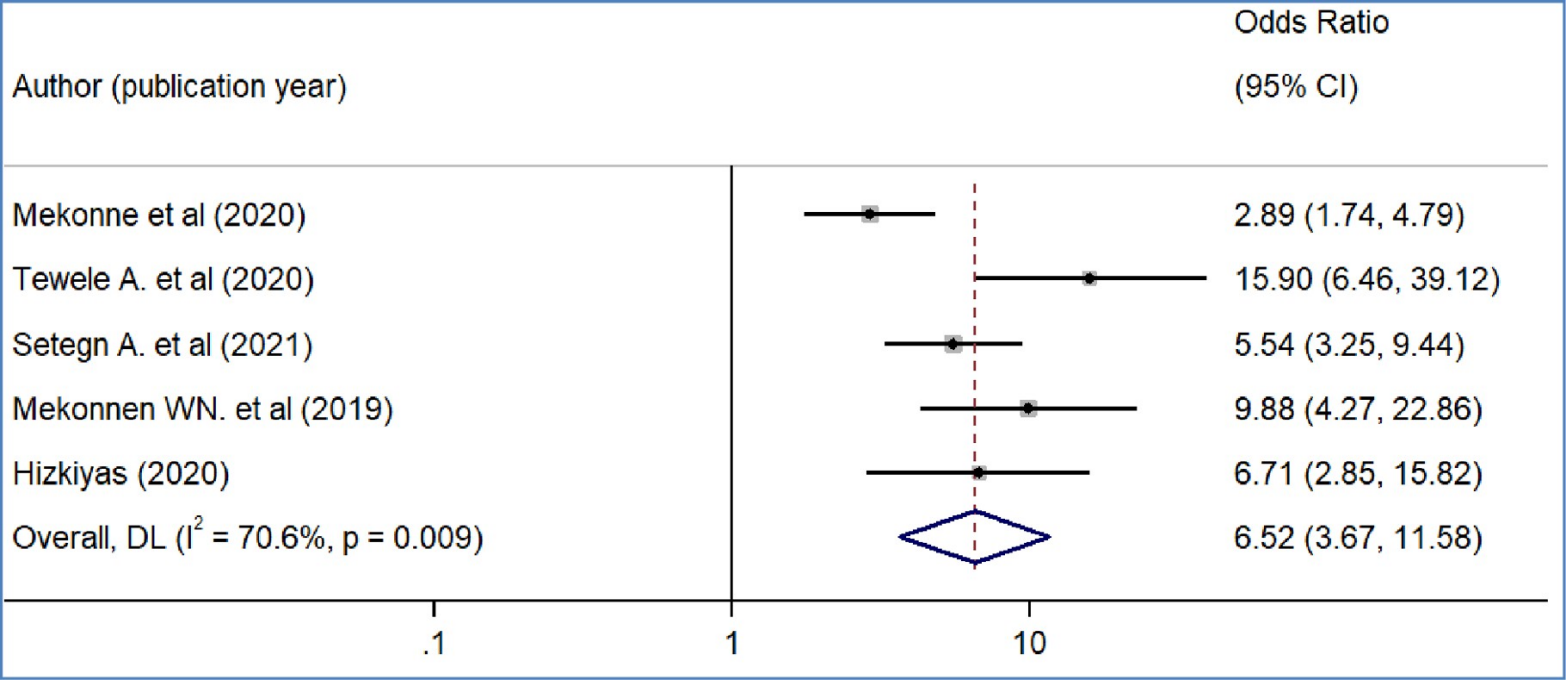
The pooled odds ratio of the association between willingness to pay for SHI and monthly salary in Ethiopia, 2022.

**Fig 8:**
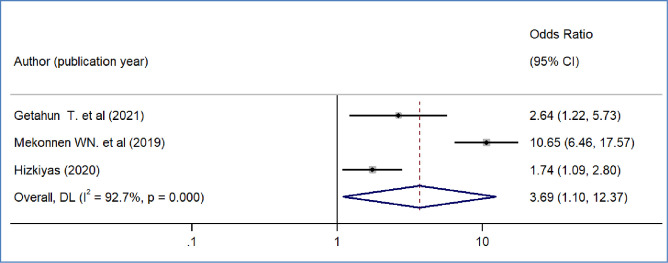
The pooled odds ratio of the association between willingness to pay for SHI and perceived quality of service in Ethiopia, 2022.

**Fig 9:**
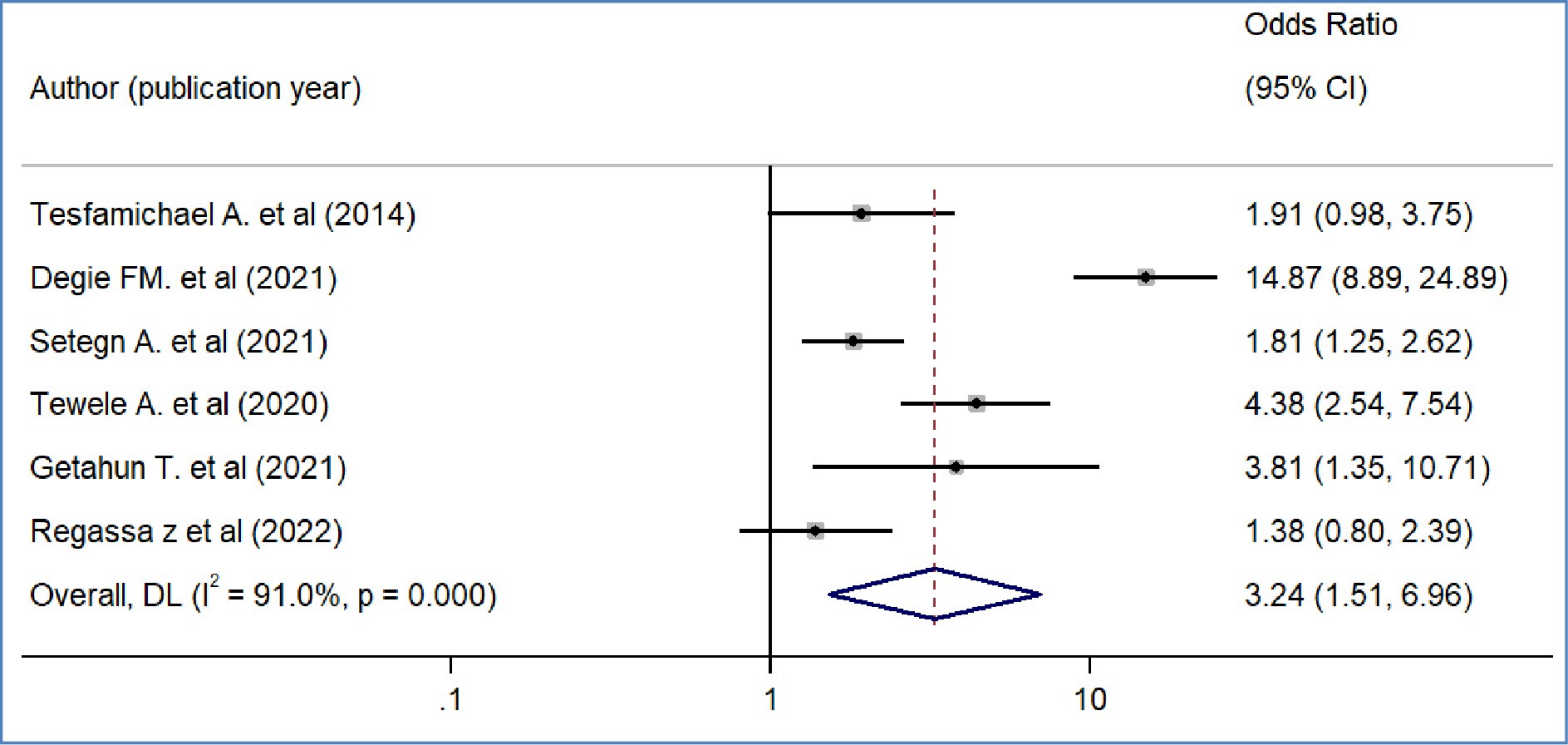
The pooled odds ratio of the association between willingness to pay for SHI and problem paying bills in Ethiopia, 2022.

**Fig 10:**
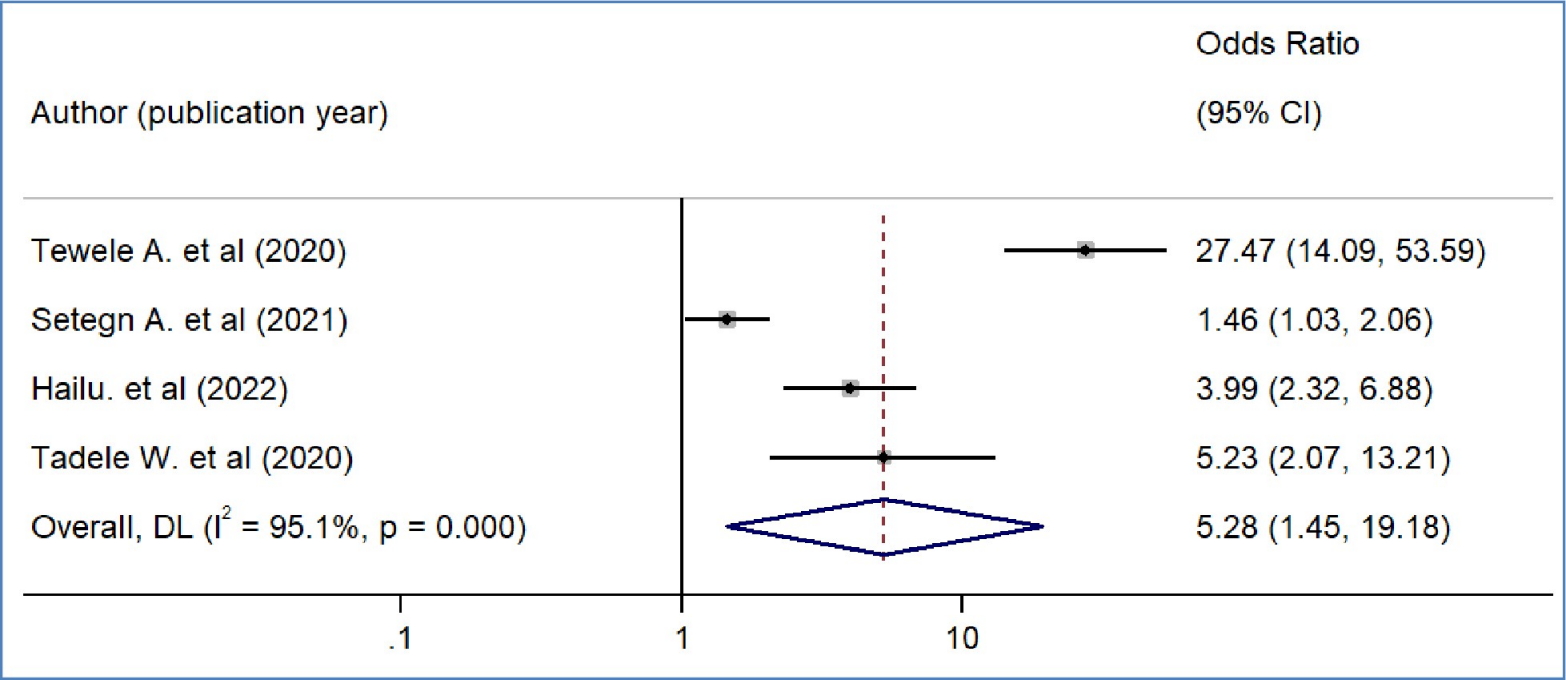
The pooled odds ratio of the association between willingness to pay for SHI and attitude towards the SHI program in Ethiopia, 2022.

**Fig 11:**
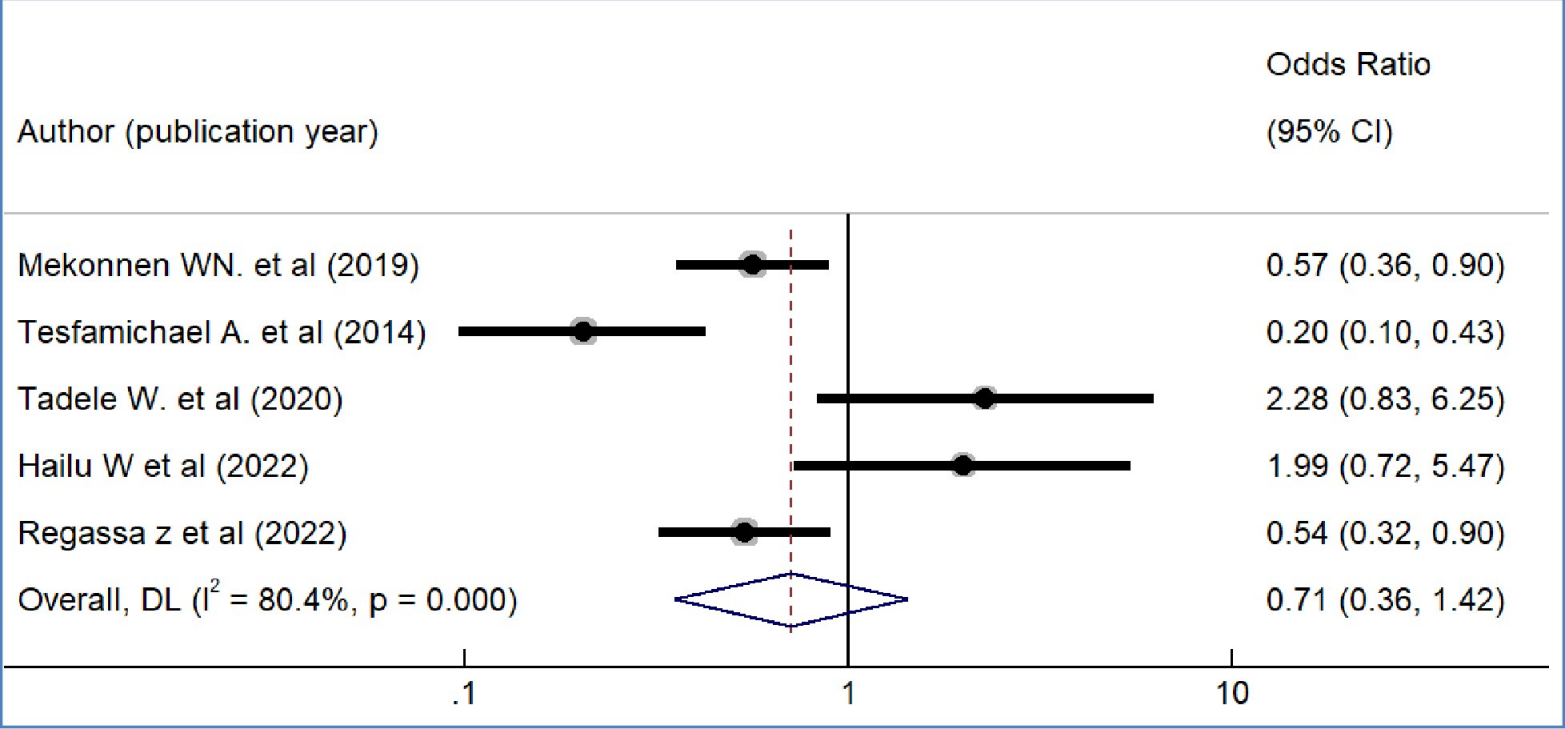
The pooled odds ratio of the association between marital status and willingness to pay for SHI in Ethiopia, 2022.

We included nine primary studies [14–18, 20, 21, 32, 36] conducted on Ethiopian Civil servants to determine the association between workers’ awareness of the SHI scheme and the WTP for the SHI scheme. The results of a meta-analysis of nine studies indicated that the odds of WTP for the SHI scheme were 3.09 times higher among workers who had awareness of the SHI scheme than workers who had no awareness of the SHI program (OR = 3.09, 95%CI: 2.12–4.48)(Fig 4). We also found the pooled analysis of six studies [13, 16, 19, 31, 32, 39] showed that workers’ perception of the quality of services in public facilities has a statistically significant impact on the pooled magnitude of WTP for the SHI scheme in Ethiopia. The meta-analysis of six studies revealed that the odds of WTP for the SHI scheme were almost four times likely to pay among workers who perceived there is a better quality of services in public health facilities than their counterparts(OR = 4.2, 95% CI: 1.97, 8.95), p-value < 0.001(Fig 5). In our meta-analysis, the pooled effects of four studies [14, 17, 18, 31] revealed that participants’ educational status was significantly associated with the WTP for the SHI scheme (OR = 5.52, 95% CI: 4.42, 7.17) (Fig 6). Five studies [14, 16, 17, 20, 31] were used to determine the association between respondents’ monthly salary and WTP for the SHI scheme among civil servants in Ethiopia. This meta-analysis indicated that respondents who had a high monthly salary were almost seven times more likely to pay for the SHI scheme compared to those who earned a lower monthly salary(OR = 6.52, 95% CI: 3.67, 11.58) (Fig 7). In this meta-analysis, we included three primary studies [20, 30, 31] conducted among different Ethiopian civil servants to investigate the association between family size and WTP for the SHI scheme. According to the meta-analysis findings, the odds of paying for the SHI scheme among respondents who had large family members were 3.69 times more likely compared to study participants who had fewer family (AOR 3.69, 95% CI: 1.10, 12.36) (Fig 8). Six studies [14, 16, 18, 21, 30, 37] were included in the meta-analysis to determine the association between the difficulty of paying medical bills and WTP for the SHI scheme. The odds of paying for the SHI program among those who had difficulty paying bills were almost three times more likely higher compared to their counterparts(AOR 3.24, 95% CI: 1.51, 6.96) (Fig 9). We used four primary studies to explore the association between workers’ attitudes towards the SHI program and WTP for the SHI scheme among public servants in Ethiopia [14, 16, 33, 36]. According to the findings of a meta-analysis, workers who had favourable attitudes were almost five times more likely to pay for the SHI scheme compared to their counterparts (OR = 5.28, 95%CI: 1.45–19.18) (Fig 10). We used five primary studies to explore the association between respondents’ marital status and WTP for the SHI scheme among Ethiopian workers [18, 31, 33, 36, 37]. According to a meta-analysis of five primary studies, workers’ marital status was not significantly associated with the WTP for the SHI scheme among public workers in Ethiopia (OR = 0.71, 95%CI: 0.36–1.42) (Fig 11).

## Discussion

Financial risk protection is one of the pillars of universal health coverage. The lack of adequate healthcare financing and access to basic health services is still a problem in Ethiopia [38]. Introducing prepayment schemes such as community-based health insurance and SHI reduces large out-of-pocket payments for health care and overcomes financial barriers to accessing health care (33). Thus, this systematic review and meta-analysis intended to examine the pooled magnitude of willingness to pay for the SHI scheme and its associated factors among public servants in Ethiopia. The pooled magnitude of willingness to pay for the SHI scheme among public servants in Ethiopia was found to be 49.62% (95% CI: 36.41–62.82). This finding is supported by a report from South Sudan, 52% [40], and Gondar town, Ethiopia, 62% [14]. However, it is less than the studies done in Bangladesh, 80.1% [41], Saudi Arabia, 77.9% [42], Vietnam, 71.6% [43], SierraLeone,94% [44], Nigeria, 82% [45], Zambia,80% [46] and higher than studies from Malaysia,33.2% [47]. This could be because of the difference in the study period, study area, level of awareness about the SHI program, quality of services provided in the public health facilities, and the perception of public servants towards the SHI scheme.

This meta-analysis revealed that those public servants who had a degree and above educational status had higher odds of paying for the SHI scheme than their counterparts. This is almost similar to previous studies, which indicated public servants with a degree and above were more likely to pay for the SHI scheme [18, 42, 44, 48, 49]. This might be explained by those who had increased educational levels having a better understanding of the principles of the SHI program and the benefit of joining the SHI to overcome catastrophic health expenditure. In addition, we found that participants who had large family sizes were more likely to pay for the SHI scheme than those who had few family sizes. This is similar to studies conducted in Bangladesh [41], and Nigeria [45]. This can be explained by the excessive out-of-pocket expenses from medical bills that large family members’ respondents faced at a time of occurrences of unpredictable illnesses that lead to catastrophic health expenditure. In this review, we found respondents’ monthly income (salary) was found to be significantly associated with the WTP for the SHI scheme. This is evidenced by a similar finding from India [49], Nigeria [48], and Sierra Leone [44], in which having an improved monthly salary has a significant positive association with WTP for the SHI scheme. This might be explained by individuals who have a high monthly salary and can afford the SHI scheme contribution at any price.

In this Meta-analysis and review, we found that participants who heard about the SHI program were more likely to pay for the scheme than those who had no awareness of the SHI program. This is in line with previous findings from studies done in India [49], and Vietnam [43]. This might be explained by those who heard about the principles of the SHI scheme might know the benefit of supporting the SHI program to halt the catastrophic out-of-pocket health expenditure. We observed public workers who perceived there is a good quality of services in public health facilities were more likely to pay for the SHI scheme than their counterparts. This is in line with previous findings from studies done in Indonesia [50], Saudi Arabia [51, 52] and Kenya [53]. This might be explained by the existence of good quality health services in public health facilities that assist the respondents in getting equitable, effective, and efficient services that motivate them to pay for the SHI scheme. Furthermore, we found that participants who had favourable attitudes towards the SHI scheme were almost five times higher odds of paying for the SHI scheme than their counterparts. This evidence is supported by findings from previous studies in Saudi Arabia [51], and Sudan [54]. This might be due to having a favourable attitude which will increase individuals’ preference to choose and use the SHI scheme.

In this systematic review, we found that participants who had difficulty paying bills had almost four times higher odds of paying for the SHI scheme than their counterparts. This is supported by findings of the study done in Nigeria [55]; in which suffering from covering the cost of illness has a significant positive association with willingness to pay for the SHI scheme. This might be explained by individuals who suffer from the difficulty of paying medical bills have a fear of catastrophic out-of-pocket healthcare expenditure at service delivery points and are more likely to pay for the SHI scheme. Our study has several limitations. Firstly, we didn’t get data from all regions of the country and this might affect the representativeness of our study. Secondly, we observed remarkable inter-study heterogeneity. Third, the difference in the categorization of some study variables was a great challenge while conducting the pooled odds ratio.

## Conclusion

The result of this meta-analysis and systematic review showed that the magnitude of willingness to pay for Social Health insurance was low in Ethiopia. Willingness to pay for Social Health Insurance was significantly associated with monthly salary, educational status, family size, the difficulty of paying medical bills, quality of healthcare services, attitude, and awareness of respondents about the SHI scheme. Hence, it’s recommended to conduct awareness creation through on-job training about the SHI program benefit packages and principles to improve the willingness to pay for the scheme among public servants in Ethiopia.

## Supporting information

S1 Checklist PRISMA checklist. (DOCX)

S1 Table Summary of search results from PubMed, Google Scholar, and other databases. (DOCX)

S2 Table Quality assessment of studies using the modified Newcastle Ottawa scale for cross sectional studies for systematic review meta-analysis of willingness to pay for Social Health Insurance and associated factors among Public Civil servants in Ethiopia. (DOCX)

## Abbreviations

CBHI: Community-based Health Insurance
CI: Confidence Interval
LMIC: Low to Medium-Income Countries
NOS: Newcastle-Ottawa Scale
OR: Odd Ratio
UHC: Universal Health Coverage
OOP: Out-of-Pocket Payment
PRISMA: Preferred Reporting Items for Systematic Review and Meta-Analysis
SHI: Social Health Insurance
WHO: World Health Organization
