# Supplementary material for: Willingness to pay for Social Health Insurance and associated factors among Public Civil Servants in Ethiopia: A systematic review and meta-analysis
Source: PLoS One. 2024 Feb 9;19(2):e0293513. doi: 10.1371/journal.pone.0293513 (PMC10857707; doi:10.1371/journal.pone.0293513)
Supplement: S1 Table — (DOCX) [file pone.0293513.s002.docx]

**(Supplementary 2)Quality assessment of studies using the modified Newcastle Ottawa scale for cross-sectional studies for systematic review meta-analysis of willingness to pay for Social Health insurance and associated factors among workers in Ethiopia**

|  | **Methodological quality ( 5 stars)** | | | | **Comparability ( 2 stars)** | **Outcome ( 3 stars)** | |  |
| --- | --- | --- | --- | --- | --- | --- | --- | --- |
| **Studies** | Representativeness of the sample(*) | Samples size(*) | Non- respondents(*) | Ascertainment of the exposure(**) | Confounding factors controlled(**) | Assessment of outcome(**) | Statistical test(*) | Total quality  score  (**10*)** |
| Abebaw B. et al | * | * | * | - | ** | ** | * | ********(8) |
| Zemene. et al | * | * | - | - | ** | ** | * | *******(7) |
| Tewele A. et al. | * | * | - | - | ** | ** | * | *******(7) |
| Lasebew Y. et al | * | * | - | - | ** | ** | * | *******(7) |
| Degie FM. et al | * | * | - | - | ** | ** | * | *******(7) |
| Tesfamichael A. et al | * | * | - | - | ** | ** | * | *******(7) |
| Mekonne A. et al | * | * | - | - | ** | ** | * | *******(7) |
| Getahun T. et al | * | * | - | - | ** | ** | * | *******(7) |
| Gessese TA. et al | * | * | * | - | ** | ** | * | ********(8) |
| Setegn A. et al | * | * | - | - | ** | ** | * | *******(7) |
| Gidey. et al | * | * | - | - | ** | ** | * | *******(7) |
| Yeshiwas S. et al | * | * | - | - | ** | ** | * | *******(7) |
| Mekonnen WN. Et al | * | * | - | - | ** | ** | * | *******(7) |
| Hizkiyas | * | * | - | - | ** | ** | * | *******(7) |
| Mulatu B. et al | * | * | - | - | - | ** | * | *****(5) |
| Regassa Z. et al | * | * | - | - | ** | ** | * | *******(7) |
| Hailu et al | * | * | * | - | ** | ** | * | ********(8) |
| Tadele W. et al | * | * | - | - | ** | ** | * | *******(7) |
| Obsie A. et al | * | * | - | - | ** | ** | * | *******(7) |
| Terefe | * | * | * | - | ** | ** | * | ********(8) |
