# Supplementary material for: Willingness to pay for Social Health Insurance and associated factors among Public Civil Servants in Ethiopia: A systematic review and meta-analysis
Source: PLoS One. 2024 Feb 9;19(2):e0293513. doi: 10.1371/journal.pone.0293513 (PMC10857707; doi:10.1371/journal.pone.0293513)
Supplement: S2 Table — (DOCX) [file pone.0293513.s003.docx]

Supplementary file 2: Table S1. Literature Search strategies for willingness to pay for Social Health Insurance among public Civil servants in Ethiopia

| S.N. | Database | Search strategy | Search results |
| --- | --- | --- | --- |
| 1 | PubMed | ((((((((Willingness[All Fields] AND Pay[All Fields]) OR Acceptance[All Fields]) OR Demand[All Fields]) AND (Social[All Fields] AND ("insurance, health"[MeSH Terms] OR ("insurance"[All Fields] AND "health"[All Fields]) OR "health insurance"[All Fields] OR ("health"[All Fields] AND "insurance"[All Fields])))) AND (Associated[All Fields] AND factors[All Fields])) OR Determinants[All Fields]) OR Predictors[All Fields]) AND ("public"[All Fields] AND Civil[All Fields] AND Servants[All Fields])) AND ("ethiopia"[MeSH Terms] OR "ethiopia"[All Fields]) | 639 |
| 2 | Google scholar | "Willingness to pay" or "Acceptance" or " Demand" and " Social Health Insurance" and "determinants" or "associated factors" or " predictors" and “Ethiopia" | 35 |
| 3 | Other databases | "Willingness to pay" or "Acceptance" or " Demand" and " Social Health Insurance" and "determinants" or "associated factors" or " predictors" and “Ethiopia" | 219 |
|  | Total |  | 893 |
